# The Clinical and Prognostic Significance of Ribonucleotide Reductase Subunits RRM1 and RRM2 mRNA Levels in Patients with Chronic Lymphocytic Leukemia

**DOI:** 10.1007/s44228-023-00033-x

**Published:** 2023-02-22

**Authors:** Sevastianos Chatzidavid, Christina-Nefeli Kontandreopoulou, Panagiotis T. Diamantopoulos, Nefeli Giannakopoulou, Panagiota Katsiampoura, Christos Stafylidis, Georgios Dryllis, Marie-Christine Kyrtsonis, Maria Dimou, Panayiotis Panayiotidis, Nora-Athina Viniou

**Affiliations:** 1 Hematology Unit, First Department of Internal Medicine, Laikon General Hospital, National and Kapodistrian University of Athens, 17 Agiou Thoma Street, Athens, Greece; 2 Hematology Section of the First Department of Propaedeutic Internal Medicine, Laikon University Hospital, Athens, Greece; 3 Department of Hematology and Bone Marrow Transplantation Unit, National and Kapodistrian University of Athens, School of Medicine, Laikon General Hospital, Athens, Greece

**Keywords:** Chronic lymphocytic leukemia, Ribonucleotide reductase, mRNA level quantification, Promoter methylation

## Abstract

Ribonucleotide Reductase (RNR) converts ribonucleotides to deoxyribonucleotides required for DNA replication and repair. RNR consists of subunits M1 and M2. It has been studied as a prognostic factor in several solid tumors and in chronic hematological malignancies, but not in chronic lymphocytic leukemia (CLL). Peripheral blood samples were collected from 135 CLL patients. M1/M2 gene mRNA levels were measured and expressed as a RRM1-2/GAPDH ratio. M1 gene promoter methylation was studied in a patients’ subgroup. M1 mRNA expression was higher in patients without anemia (*p = *0.026), without lymphadenopathy (*p = *0.005) and 17p gene deletion (*p = *0.031). Abnormal LDH (*p = *0.022) and higher Rai stage (*p = *0.019) were associated with lower M1 mRNA levels. Higher M2 mRNA levels were found in patients without lymphadenopathy (*p = *.048), Rai stage 0 (*p = *0.025) and Trisomy 12 (*p = *0.025). The correlation between RNR subunits and clinic-biological characteristics in CLL patients demonstrate RNR’s potential role as a prognostic factor.

## Introduction

**Fig. 1:**
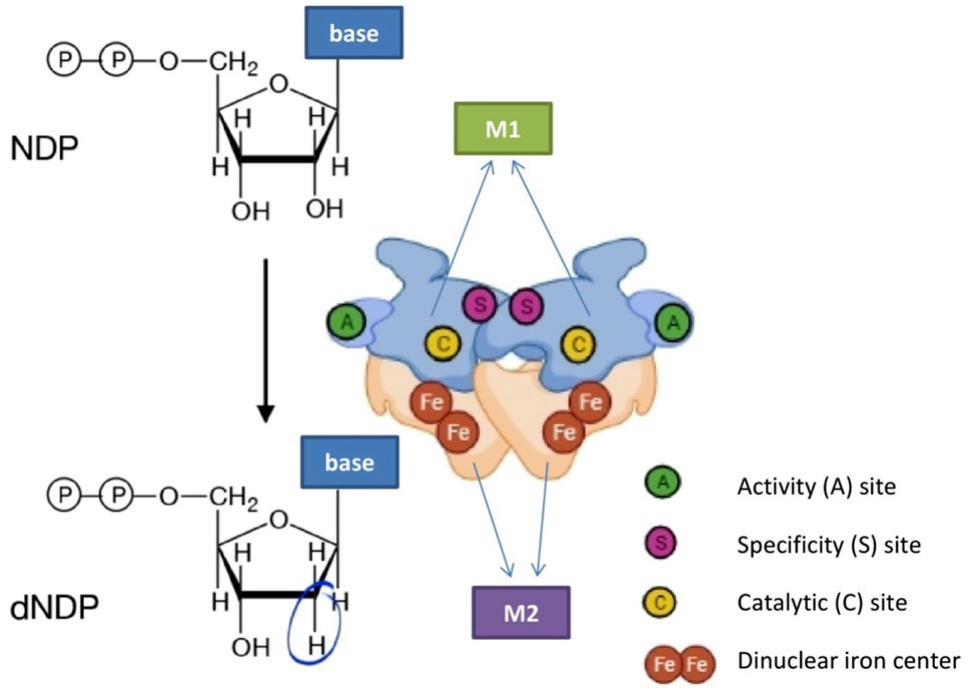
Ribonucleotide reductase structure and function. *NDP* nucleoside diphosphate, *dNDP* deoxy-nucleoside diphosphate, *M1* ribonucleotide reductase subunit 1, *M2* ribonucleotide reductase subunit 2. M1 subunit contains the catalytic site and two different allosteric sites whereas M2 subunit contains a di-iron cofactor and tyrosyl radical crucial for RNR activity. The subunits form hetero-oligomers with the active enzyme adopting a quaternary state

Ribonucleotide reductase (RNR) is responsible for converting ribonucleotides to deoxyribonucleoside triphosphates (dNTPs) and maintaining the dNTP pool necessary for DNA synthesis [1]. Human RNR consists of an M1 subunit encoded by the RRM1 gene (ribonucleotide reductase regulatory subunit M1), and an M2 subunit encoded by the RRM2 (ribonucleotide reductase regulatory subunit M2) and p53R2 (ribonucleotide reductase M2 B) genes, which encode two different M2 isoforms [2]. The M1 subunit contains the catalytic site and two different allosteric sites, whereas M2 contains a di-iron cofactor and tyrosyl radical crucial for RNR activity. The subunits form hetero-oligomers with the active enzyme adopting a quaternary state [3] (Fig. 1).

One of the most important regulation mechanisms of RNR is the allosteric regulation which is dependent on its substrates and products. Another essential regulation imposed on RNR is exerted by the cell cycle. The status of RNR activity and DNA synthesis have been shown to be closely correlated. The total dNTP pool reaches its peak size during the S-phase to support nuclear DNA replication, with a tenfold lower nadir in G_0_/G_1_, when dNTPs are needed only for DNA repair and mitochondrial DNA synthesis [4, 5]. Failure of cells to maintain a proper dNTP pool can be harmful, leading to DNA breaks, mutagenesis, and cell apoptosis [6]. RRM2 expression is regulated in a cell cycle-specific manner whereas RRM1 expression remains relatively stable in proliferating cells [3].

One of the most defining aspects of cancer biology is uncontrolled proliferation which, in turn, requires an increased quantity of dNTPs. Previous studies showed that the RNR activity correlated, in some cases, with cancer growth rate, and large differences in activity were recorded between slow- and fast-growing neoplastic cells [6]. Moreover, RRM2 gene overexpression has been found in breast, ovarian, gastric, bladder, colorectal cancers, and melanoma. RRM2 gene expression was found to be correlated with tumor grade in both breast and epithelial ovarian malignancies and poor overall survival in melanoma. In a similar pattern, the RRM1 gene was overexpressed in brain and central nervous system cancer, lung cancer and sarcoma [7–15].

Chronic lymphocytic leukemia (CLL) is characterized by gradual accumulation of monoclonal and functionally incompetent lymphocytes in the peripheral blood, lymph nodes, and the spleen. CLL cells are likely derived from pre-germinal center mature CD5 + B cells or post-germinal center B cells [16–18]. The kinetic balance between apoptosis and proliferation is impacted by increased expression of the antiapoptotic protein B cell leukemia/lymphoma 2 (BCL2), genetic features (eg, IGHV mutation status), breakpoint cluster region protein  signaling, and interactions with the tumor microenvironment or antigens [19, 20]. Previous studies have revealed the complex heterogeneity of CLL cells by demonstration of genomic aberrations in *TP53*, *SF3B1*, *BIRC3*, *NOTCH1*, and *ATM* that were found to be critical in the progression of the disease [21, 22].

CLL cells are characterized by an indolent nature and remain mainly in the G_0_ and G_1_ phase of the cell cycle. Previous studies have shown that RNR is regulated in CLL cells during both replication and DNA repair after cytotoxic treatment. More specifically, it was reported that a dose-dependent increase in protein levels of the M2 subunit and stable M1 subunit protein levels were observed. The changes in protein levels were correlated with the RRM1 and RRM2 mRNA levels [23]. Our present study looks at the correlation between RNR expression and clinical features in CLL. Moreover, our findings of a potential link between RNR expression and prognostic factors is intriguing and suggests a potential role of this protein in the pathobiology of this disease.

## Materials and Methods

### Patient Characteristics and Blood Sampling

We enrolled 135 Greek patients with immunophenotypically confirmed CLL at the time of sample collection. The diagnosis of CLL was based on the 2018 International Workshop on Chronic Lymphocytic Leukemia (iwCLL) criteria [24]. Basic laboratory and clinical parameters during the period of diagnosis, along with the Rai and Binet stages were recorded for each patient. Time to first line treatment was calculated from diagnosis to the first documented treatment administration. Time between the first and second line treatment was calculated from the last documented cycle of the first line to the first documented cycle of the second line treatment. Overall survival was calculated from diagnosis to last follow-up or death.

The study protocol was approved by the institutional ethics committee, ensuring compliance with the Helsinki Declaration, as revised in 2008. All participants provided written informed consent.

### RRM1 and RRM2 mRNA Level Quantification

After sample collection, RNA extraction and reverse transcription were performed using standard protocols. RRM1 and RRM2 mRNA levels were determined by a quantitative real-time polymerase chain reaction (RT-PCR). Quantification of the RRM1 gene promoter methylation was conducted in a randomly selected subgroup of 100 samples. Samples from twenty healthy adults matched for gender and age were used as a control group.

Total RNA from peripheral blood cells was extracted using the Purelink RNA mini kit (Invitrogen, Carlsbad, CA, USA). Reverse transcription was performed using an MMLV-derived reverse transcriptase (MMLV RT, Invitrogen, Carlsbad, CA, USA) according to the manufacturer’s recommendations. For the RT-PCR, the CFX-96 real-time PCR system (Bio-Rad Laboratories, Hercules, CA, USA) was used. Each reaction was set up in a 10-μl total volume including 2 μl cDNA from each sample and a TaqMan Universal PCR mix (Applied Biosystems, Waltham, MA, USA). For assays containing the ABI Gene Expression Assay primer/probe sets, 1 μl primer and probe mix (20X concentration) was added to the reaction. The PCR amplification was performed with a 96-well plate using default conditions. During the PCR, the probe was cleaved by the Taq DNA polymerase releasing the 5ʹ end reporter fluorescence dye (FAM/MGB). Its fluorescence was detected using the CFX-96 Sequence Detection System. The values of gene expression were normalized by using the glyceraldehyde-3-phosphate dehydrogenase (GAPDH) expression level. The standard curve method was used to calculate the relative quantitation of RNR and GAPDH transcripts, and RNR mRNA levels were expressed as a ratio of RNR to GAPDH transcript levels. The following assays were used: RRM1:Hs01040698_m1, RRM2: Hs00357247_g1 and GAPDH: Hs02786624_g1 (Applied Biosystems, Waltham, MA, USA).

### RRM1 Gene Promoter Methylation Level Assessment

The EpiTect Methyl qPCR Array (SA Bioscience, Qiagen, Hilden, Germany) was used under manufacturer’s instructions to quantify *RRM1* promoter methylation levels. The following digestion reactions were performed: no enzyme, methylation-dependent enzyme, methylation-sensitive enzyme, and methylation-sensitive and -dependent enzymes. A SYBR green-based RT-PCR was carried out with a CFX96 RT-PCR system (Bio-Rad Laboratories, Hercules, CA, USA) using pre-designed primers that flank the loci [(EPHS102129-1A), CpG island location: Chr11: 4115689–4,116489 (300 bp)]. The level of the RRM promoter methylation 1 was calculated and reported as the percentage of unmethylated (F_UM_) and methylated (F_M_) fraction. The optimal cutoff value for the differentiation of “methylated” versus”unmethylated” status of the RRM1 gene promoter was defined as > 60% for F_M_ and < 30% for F_UM,_ according to the manufacturer's specifications.

### Statistical Analysis

The Mann–Whitney and Kruskal–Wallis tests were used for the comparison of RRM1/GAPDH, RRM2/GAPDH expression and methylation levels between two or more than two groups, respectively. Spearman correlation coefficients were used to explore the association of two continuous variables. All reported p values are two-tailed. Statistical significance was set at *p* < 0.05 and analyses were conducted using SPSS statistic (version 22).

## Results

### Patient Characteristics

**Fig. 2:**
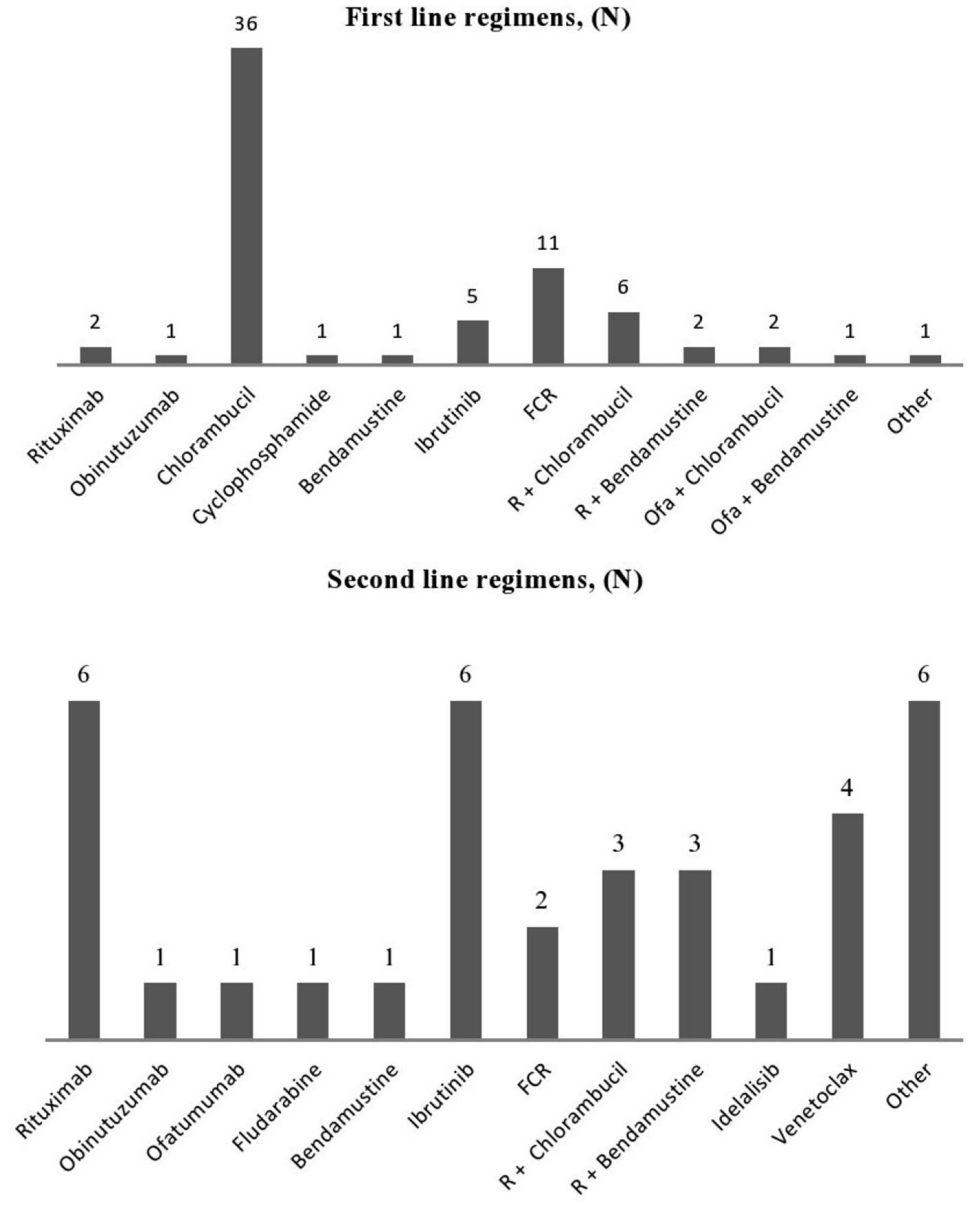
First and second line treatment regimens. *FCR* fludarabine + cyclophosphamide + rituximab, *R* rituximab, *Ofa* ofatumumab

A total of 135 patients were enrolled, of which 56.3% were female. The median age at diagnosis was 64 years (IQR: 55–73 years). The majority of the patients (62.2%) had their blood collected before treatment. The median overall time of follow-up was 6.66 years (IQR: 3.47–11.13 years), and the median time from diagnosis to first line treatment was 23.1 months (IQR: 5.8–56.5 months). Sixty-nine patients (51.1%) received at least one line of treatment, whereas thirty-five (25.9%) had a second line treatment. Furthermore, 73.1% of the patients were Binet stage A and 82.3% were RAI stage 0 or I. The IGHV mutation status was available in 25/135 patients. Results for FISH analyses regarding trisomy 12, 17p and 11q deletions were available in 23/135, 82/135 and 42/135 patients, respectively. The patient characteristics and the treatment regimens are shown in Table 1 and Fig. 2, respectively.

**Table 1:** Patient characteristics

Characteristics	No. of patients	%
Gender		
Male	76	56.3
Female	59	43.7
Median age of diagnosis, (IQR)	64 years, (55–73)	
Binet stage		
A	95	73.1
B	27	20.8
C	8	6.2
Rai stage		
0	63	48.5
I	44	33.8
II	16	12.3
III	4	3.1
IV	3	2.3
Presence of splenomegaly	16	12.4
Presence of lymphadenopathy	66	50.8
Median lymphocyte count, (IQR)	14,385/μL (9388.5–26,030)	
Median hemoglobin count, (IQR)	13.5 g/dL (12.6–14.65)	
Median platelet count, (IQR)	198 × 10^9^/L (160–228.5)	
Median LDH, (IQR)	211 U/L (180–290)	
Median ESR, (IQR)	10 mm/h (6–22)	
Cytogenetics		
17p −	3	3.7
11q −	2	4.8
Trisomy 12	5	21.7
Normal	12	8.9
IGHV gene mutation status		
Unmutated	13	52
Mutated	12	48
Patients received first line treatment	69	51.1
Patients received second line treatment	35	25.9
Correlation between blood collection and treatment line		
Treatment-naïve patients	84	62.2
After 1 Line	27	20
After 2 Lines	15	11.1
After 3 Lines	9	6.7
RRM1 mRNA expression, median (IQR)	0.04 (0–0.09)	
RRM2 mRNA expression, median (IQR)	0.01 (0–0.1)	
M1 protein Western blot, median (IQR)	1.19 (0.98–1.47)	
RRM1 promoter methylation, unmethylated fraction (%), median (IQR)	80.67 (0–36.48)	
RRM1 promoter methylation, methylated fraction (%), median (IQR)	21 (0–36)	

*No.* number, *SD* standard deviation, *IQR* interquartile range, *LDH* lactate dehydrogenase, *ESR* erythrocyte sedimentation rate, *17p-* deletion 17p, *11q-* deletion 11q, *IGVH* immunoglobulin variable heavy chain, *RRM1* ribonucleotide reductase regulatory subunit M1 gene, *RRM2* ribonucleotide reductase regulatory subunit M2 gene

### RRM1 and RRM2 mRNA Levels and Patient Parameters

The median expression of RRM1/GAPDH was 0.04 (IQR: 0–0.09) and of RRM2/GAPDH was 0.01 (IQR: 0–0.1). As shown in Table 2, RRM1 mRNA levels were higher in patients without anemia (*p = *0.026) or lymphadenopathy (*p = *0.005). Erythrocyte sedimentation rate (ESR) values greater than 30 mm/h (*p = *0.018), LDH levels above normal (*p = *0.022) and higher Rai stage (*p = *0.019) were associated with lower RRM1 mRNA levels. In addition, 17p deletion was found to be associated with higher RRM1 mRNA levels (*p = *0.031).

**Table 2:** RRM1 mRNA expression and patient parameters

	RRM1 mRNA expression, median (IQR)	*p*
Presence of lymphadenopathy		
Yes	0.02 (0–0.07)	0.005
No	0.06 (0.01–0.25)	
Rai score		
0	0.06 (0.01–0.24)	0.019^a^
I	0.02 (0–0.06)	
II/ III/ IV	0.03 (0–0.12)	
TP53 gene deletion (detected by FISH)		
Yes	0.55 (0.08–20.5)	0.031
No	0.05 (0–0.09)	
Hemoglobin level (g/dL)		
≤ 11	0 (0–0.03)	0.026
> 11	0.05 (0–0.09)	
ESR (mm/h)		
≤ 30	0.05 (0–0.16)	0.018
> 30	0 (0–0)	
LDH (U/L)		
≤ 225	0.06 (0.01–0.1)	0.022
> × 1 ULN	0.01 (0–0.08)	

*RRM1* ribonucleotide reductase regulatory subunit M1 gene, *IQR* interquartile range, p: p-value, TP53: tumor protein 53, *FISH* fluorescence in situ hybridization, *ESR*: erythrocyte sedimentation rate, *LDH* lactate dehydrogenase, *ULN* upper limit of normal

^a^Kruskal–Wallis test. Mann–Whitney test and Kruskal–Wallis test were used for the comparison of RRM1/GAPDH expression between two or more than two groups respectively. Spearman correlations coefficients were used to explore the association of two continuous variables. All reported *p* values are two-tailed. Statistical significance was set at *p* < 0.05

More interestingly, among patients whose blood was sampled before treatment, it was found that higher Rai score (*r* = − 0.30; *p = *0.005) and less time between the two lines of treatment (*r* = 0.95; *p = *0.050) were significantly associated with lower expression of RRM1/GAPDH.

Significantly higher RRM2 mRNA levels were reported in patients without lymphadenopathy (*p = *0.048) and Rai stage 0 (*p = *0.025). Moreover, the levels of RRM2 mRNA were higher in cases with trisomy 12 (*p = *0.025), (Table 3). RRM2 mRNA expression was not associated with deletion 17p in our study (*p = *0.931).

**Table 3:** RRM2 mRNA expression and patient parameters

	RRM2 mRNA expression, median (IQR)	*p*
Presence of lymphadenopathy		
Yes	0.01 (0–0.08)	0.048
No	0.04 (0–0.22)	
Rai score		
0	0.05 (0–0.23)	0.025 +
I	0 (0–0.06)	
II/ III/ IV	0.02 (0–0.35)	
Trisomy 12		
Yes	0.15 (0.06–0.19)	0.025
No	0 (0–0.01)	

Mann–Whitney test and Kruskal–Wallis test were used for the comparison of RRM2/GAPDH expression between two or more than two groups respectively. Spearman correlations coefficients were used to explore the association of two continuous variables. All reported *p* values are two-tailed. Statistical significance was set at *p* < 0.05

*RRM2* ribonucleotide reductase regulatory subunit M2 gene, *IQR* interquartile range, *p* *p* value, + : Kruskal–Wallis test

### RRM1 Gene Promoter Methylation Levels and Patient Parameters

Α “methylated” status (F_Μ_) of RRM1 gene promoter was correlated with the presence of lymphadenopathy (*p = *0.016). Lymphadenopathy was also correlated with higher levels of methylation (28.8% versus 20.9%, *p = *0.039). A “methylated” status was correlated with about 4 times lower levels of RRM1 mRNA compared to “unmethylated” status (0.0123 versus 0.0465, *p = *0.014), and with about 10 times lower levels of RRM2 mRNA (0.0038 versus 0.0397 for “unmethylated” status, *p = *0.027) (Table 4).

**Table 4:** Correlation of RRM1 gene promoter methylation status and RRM1/RRM2 mRNA expression

	RRM1 mRNA expression, median	*p*	RRM2 mRNA expression, median	*p*
Methylation status of RRM1 gene promoter				
Methylated	0.0123	0.014	0.0038	0.027
Unmethylated	0.0465		0.0397	

*RRM1* ribonucleotide reductase regulatory subunit M1 gene, *RRM2* ribonucleotide reductase regulatory subunit M2 gene

## Discussion

### RRM1 mRNA Levels and Patient Parameters

From this study it appears that high RRM1 mRNA levels are associated with findings that could reflect a profile of favorable prognosis and disease burden.

Previous reports have shown that RRM1 mRNA in parallel with M1 protein levels remained constant, not only during replication in resting CLL cells, but also during DNA repair after cytotoxic treatment, although total RNR protein expression was increased in the latter [23]. A hypothesis that may incorporate our above findings is that a CLL cell with high RRM1 mRNA expression and, therefore, M1 protein expression, in turn presents increased total RNR expression under certain conditions. This could lead to an improved capability of the cell to maintain the important dNTP pool and preserve genomic stability in the early course of the disease, reflecting a phenotype of a favorable prognosis. Moreover, the fact that RRM1 expression remains relatively stable, both at non proliferating CLL cells and after cytotoxic treatment, makes it a potential prognostic factor.

On the other hand, overexpression of RNR is frequently found in malignant cells, thereby increasing the DNA synthesis pace and contributing to the proliferative feature of the malignant cell clone and disease burden. In CLL, the Rai system uses physical examination and full blood counts to stage each patient (0-IV), which correspond to three risk groups (low 0, intermediate I-II, and high III-IV) with different predicted overall survival, and to define patients who would benefit from treatment. Increased stage reflects an increase in the total burden of leukemic lymphocytes. However, this system was developed 40–50 years ago and, as advances in CLL have improved our therapeutic approaches, the prognosis for each stage has improved dramatically, and the survival curves reported in the original publications are significantly shorter than the expected survival with modern therapy [25, 26]. Τhe correlation of RRM1 expression with the Rai stage may propose it as a potential indicator of disease burden.

However, the correlation between high RRM1 mRNA levels and 17p deletion raises some intriguing questions. In a large number of our patients, FISH analyses of 17p deletion were not available, and there was no association or trend between RRM1 mRNA expression and TP53 mutation where data were available. Τhis finding appeared in patients with more advanced disease who had received at least one line of treatment. Therefore, to avoid speculations further investigation in larger groups is needed to draw safe conclusions regarding this association.

### RRM2 mRNA Levels and Patient Parameters

In our study, higher RRM2 mRNA levels were associated with the absence of lymphadenopathy and a Rai stage of 0, which also could suggest a favorable prognosis profile, and be supported by our hypothesis regarding RRM1. In addition, M2 protein expression increases after cytotoxic treatment [23].

Τhe association between high RRM2 mRNA levels and trisomy 12 found in our study raises questions regarding the existence of a relationship and its potential clinical utilization. It is known that trisomy 12 cases comprise about 20% of CLL cases, and are often characterized by atypical morphological and immunophenotypic features, unmutated *IGVH* status, high proliferative potential with Richter transformation, and other secondary malignancies. CLL patients with trisomy 12 have an intermediate prognosis, as they require treatment earlier than patients with 13q deletion, but later than those with 11q deletion. In previous studies with the fludarabine, cyclophosphamide and rituximab (FCR) regimen, trisomy 12 patients have been reported to have a longer progression-free survival than those with other cytogenetic abnormalities, but no difference in overall survival was observed. Therefore, we can assume that although trisomy 12 CLL patients may require treatment earlier, they seem to respond better to FCR, compared to patients with other cytogenetic abnormalities [27]. In addition to the high frequency of *NOTCH1* mutations described in some previous studies, new genes and pathways involved in trisomy 12 cases were identified, including the checkpoint inhibitor NT5E and the NFAT signaling pathway, which could be targets for novel therapeutic agents [28, 29]. In our study, among five patients with trisomy 12, none carried 17p deletion, and all received one line of treatment during their disease course. As before, the relatively small number of patients for whom data were available on this particular mutation limits the ability to draw conclusions, and further testing is required.

### RRM1 Promoter Methylation Status and Its Effect

In this study, there was an association observed between RRM1 promoter hypermethylation levels and low RRM1/RRM2 mRNA expression, suggesting an epigenetic level of RNR regulation in CLL patients. Moreover, the finding of a correlation between methylation status and the presence of lymphadenopathy is very interesting, as lymphadenopathy is an important feature of CLL, and has also been related both with RRM1 and with RRM2 mRNA expression in our study, which make RNR a possible biomarker.

Previous studies have shown that extensive genetic heterogeneity exists in underlying clonal evolution in CLL patients, especially those with adverse clinical course. Despite the new emerging findings on the role of epigenetic alterations in CLL, emphasis should be given to the epigenetic mechanisms that might contribute to the disease evolution, particularly the response and resistance to treatment, in which research is still ongoing [30, 31]. Further investigation may confirm our findings and elucidate the methylation of RRM1/RRM2 promoters and their place in the complex puzzle of disease pathophysiology.

### The Role of RNR Inhibitors in CLL Treatment

RNR inhibitors have been recognized for their anti-tumor activity, and are used early in solid and hematological malignancies, either as monotherapy or as part of a multidrug regimen. As more details of RNR structure, function, and clinical importance have come to light, novel RNR inhibitors in different categories will further contribute to cancer chemotherapy, by affecting tumor initiation, progression, and therapeutic sensitivity.

Several RRM1 inhibitors have been validated for their efficacy in CLL treatment, including cisplatin, gemcitabine, and clofarabine [32, 33], with promising results. More important is the fact that another nucleoside analog, fludarabine, has been the standard of care first-line treatment of CLL as part of the FCR regimen [34], which continues to be an option for patients younger than 65 years with mutated IgVH genes [35]. Our results therefore, highlight the potential association of RRM1 expression with CLL prognosis, and emphasize a treatment strategy including specific RRM1 targeting in selected cases.

### Abnormal DNA Methylation in CLL

As many other hematological neoplasms, CLL is characterized by wide genome hypomethylation [35]. Previous studies have identified hundreds of known genes with aberrant methylation at their regulatory sites, such as the HOX gene clusters, TWIST2, DAPK1, SLIT2, or ZAP70 [36–40]. Some of the genes involved are of particular interest. For example, upregulated ZAP70 gene expression and hypomethylation in its regulatory CpG dinucleotide sites are correlated with poorer prognosis in CLL patients [40]. Another interesting finding from other studies is the identification of hypermethylation patterns in genes associated with the WNT signaling pathway in CLL patients, but a statistical correlation with clinical data was not documented [41, 42]. Other investigators reported that the hypomethylation status of the neoangiogenic factor ANGPT2 was correlated with shorter overall survival and poor prognosis in CLL patients [43]. Τhe above findings and a series of others described in several studies demonstrate the importance of DNA methylation in CLL diagnosis, prognosis and potential therapeutic targeting [44]. Previous attempts to incorporate a hypomethylating agent as monotherapy in the treatment of refractory CLL were not so successful [45]. Nevertheless, as our knowledge in this area deepens, and newer drugs come to the front, further investigation is warranted, and the combination of a hypomethylating agent with an RNR inhibitor could be an intriguing study plan in selected patients.

### Strengths and Limitations of the Study

As with many clinical studies, there are strengths and limitations that could be addressed in future research. The advantages of our study include the recording of several clinical and laboratory parameters in each patient, the blood sampling from both treatment naïve and treated CLL patients and the use of a control group. Our results demonstrated a correlation of RRM1 and RRM2 mRNA levels with clinical parameters in patients with CLL. Limitations of our study include the absence of RRM2 gene methylation and p53R2 mRNA expression assessments, and the low availability of data on certain parameters (ie FISH, parameters, IGVH mutational status) due to limited financial resources. Additional limitations are the heterogeneity in terms of the timepoints of sample collection, as well as the fact that no serial measurements of RRM1 and RRM2 levels was possible for this cohort, which could allow us investigate the dynamics of the levels of RRM1 and RRM2 before and after treatment.

### Conclusion

In the field of CLL, major progress in the last decade has led to a much better understanding of both direct and indirect mechanisms involved in CLL biology, and to the development of several new and promising treatment agents. The next stage in understanding RNR function and involvement in CLL would require larger in-depth biochemical and genetic analyses.

## Data Availability

The datasets used and/or analyzed during the current study are available from the corresponding author upon reasonable request. Citations are included in the reference list.

## References

[1] Reichard P (1985). Ribonucleotide reductase and deoxyribonucleotide pools. Basic Life Sci.

[2] Thelander L, Eriksson S, Akerman M (1980). Ribonucleotide reductase from calf thymus. Separation of the enzyme into two nonidentical subunits, proteins M1 and M2. J Biol Chem.

[3] Aye Y, Li M, Long M (2015). Ribonucleotide reductase and cancer: biological mechanisms and targeted therapies. Oncogene.

[4] Mathews CK (2006). DNA precursor metabolism and genomic stability. FASEB J.

[5] Rampazzo C, Miazzi C, Franzolin E (2010). Regulation by degradation, a cellular defense against deoxyribonucleotide pool imbalances. Mutat Res.

[6] Reichard P (1988). Interactions between deoxyribonucleotide and DNA synthesis. AnnuRevBiochem.

[7] Morikawa T, Maeda D, Kume H, Homma Y, Fukayama M (2010). Ribonucleotide reductase M2 subunit is a novel diagnostic marker and a potential therapeutic target in bladder cancer. Histopathology.

[8] Wang LM, Lu FF, Zhang SY, Yao RY, Xing XM, Wei ZM (2012). Overexpression of catalytic subunit M2 in patients with ovarian cancer. Chin Med J (Engl).

[9] Morikawa T, Hino R, Uozaki H (2010). Expression of ribonucleotide reductase M2 subunit in gastric cancer and effects of RRM2 inhibition in vitro. Hum Pathol.

[10] Lu AG, Feng H, Wang PX, Han DP, Chen XH, Zheng MH (2012). Emerging roles of the ribonucleotide reductase M2 in colorectal cancer and ultraviolet-induced DNA damage repair. World J Gastroenterol.

[11] Liu X, Zhang H, Lai L (2013). Ribonucleotide reductase small subunit M2 serves as a prognostic biomarker and predicts poor survival of colorectal cancers. ClinSci (Lond).

[12] Ma XJ, Salunga R, Tuggle JT (2003). Gene expression profiles of human breast cancer progression. Proc Natl Acad Sci USA.

[13] Aird KM, Li H, Xin F, Konstantinopoulos PA, Zhang R (2013). Identification of ribonucleotide reductase M2 as a potential target for pro-senescence therapy in epithelial ovarian cancer. Cell Cycle.

[14] Aird KM, Zhang G, Li H (2013). Suppression of nucleotide metabolism underlies the establishment and maintenance of oncogene-induced senescence. Cell Rep.

[15] Matsushita S, Ikeda R, Fukushige T (2012). p53R2 is a prognostic factor of melanoma and regulates proliferation and chemosensitivity of melanoma cells. J DermatolSci.

[16] Damle RN, Ghiotto F, Valetto A (2002). B-cell chronic lymphocytic leukemia cells express a surface membrane phenotype of activated, antigen-experienced B lymphocytes. Blood.

[17] Fais F, Ghiotto F, Hashimoto S (1998). Chronic lymphocytic leukemia B cells express restricted sets of mutated and unmutated antigen receptors. J ClinInvest.

[18] Stevenson FK, Caligaris-Cappio F (2004). Chronic lymphocytic leukemia: revelations from the B-cell receptor. Blood.

[19] Pallasch CP, Schulz A, Kutsch N (2008). Overexpression of TOSO in CLL is triggered by B-cell receptor signaling and associated with progressive disease. Blood.

[20] Messmer BT, Messmer D, Allen SL (2005). In vivo measurements document the dynamic cellular kinetics of chronic lymphocytic leukemia B cells. J Clin Invest.

[21] Nadeu F, Delgado J, Royo C (2016). Clinical impact of clonal and subclonal TP53, SF3B1, BIRC3, NOTCH1, and ATM mutations in chronic lymphocytic leukemia. Blood.

[22] Sandoval MR, Balakrishnan K, Luthra R, Keating M, Gandhi V (2014). DNA repair initiation induces expression of ribonucleotide reductase in human chronic lymphocytic leukemia cells. Leuk Lymphoma.

[23] Pflug N, Bahlo J, Shanafelt TD (2014). Development of a comprehensive prognostic index for patients with chronic lymphocytic leukemia. Blood.

[24] Autore F, Strati P, Laurenti L, Ferrajoli A (2018). Morphological, immunophenotypic, and genetic features of chronic lymphocytic leukemia with trisomy 12: a comprehensive review. Haematologica.

[25] Hallek M, Cheson BD, Catovsky D (2018). iwCLL guidelines for diagnosis, indications for treatment, response assessment, and supportive management of CLL. Blood.

[26] Sagawa M, Ohguchi H, Harada T (2017). Ribonucleotide reductase catalytic subunit M1 (RRM1) as a novel therapeutic target in multiple myeloma. Clin Cancer Res.

[27] Abruzzo LV, Herling CD, Calin GA (2018). Trisomy 12 chronic lymphocytic leukemia expresses a unique set of activated and targetable pathways. Haematologica.

[28] Del Giudice I, Rossi D, Chiaretti S (2012). NOTCH1 mutations in +12 chronic lymphocytic leukemia (CLL) confer an unfavorable prognosis, induce a distinctive transcriptional profiling and refine the intermediate prognosis of +12 CLL. Haematologica.

[29] Tsagiopoulou M, Papakonstantinou N, Moysiadis T (2019). DNA methylation profiles in chronic lymphocytic leukemia patients treated with chemoimmunotherapy. Clin Epigenetics.

[30] Kanduri M, Cahill N, Göransson H, Enström C, Ryan F, Isaksson A (2010). Richard Rosenquist; differential genome-wide array–based methylation profiles in prognostic subsets of chronic lymphocytic leukemia. Blood.

[31] Oberic L, Vaillant W, Hebraud B (2015). Clinical activity of a new regimen combining gemcitabine and alemtuzumab in high-risk relapsed/refractory chronic lymphocytic leukemia patients. Eur J Haematol.

[32] Gandhi V, Plunkett W, Bonate PL (2006). Clinical and pharmacokinetic study of clofarabine in chronic lymphocytic leukemia: strategy for treatment. Clin Cancer Res.

[33] Catovsky D, Richards S, Matutes E (2007). Assessment of fludarabine plus cyclophosphamide for patients with chronic lymphocytic leukaemia (the LRF CLL4 Trial): a randomised controlled trial. Lancet.

[34] Kahl B (2019). RIP FCR?. Hematologist.

[35] Cahill N, Rosenquist R (2013). Uncovering the DNA methylome in chronic lymphocytic leukemia. Epigenetics.

[36] Pei L, Choi JH, Liu J, Lee EJ, McCarthy B, Wilson JM, Speir E, Awan F, Tae H, Arthur G (2012). Genome-wide DNA methylation analysis reveals novel epigenetic changes in chronic lymphocytic leukemia. Epigenetics.

[37] Raval A, Lucas DM, Matkovic JJ, Bennett KL, Liyanarachchi S, Young DC, Rassenti L, Kipps TJ, Grever MR, Byrd JC (2005). TWIST2 demonstrates differential methylation in immunoglobulin variable heavy chain mutated and unmutated chronic lymphocytic leukemia. J Clin Oncol.

[38] Raval A, Tanner SM, Byrd JC, Angerman EB, Perko JD, Chen SS, Hackanson B, Grever MR, Lucas DM, Matkovic JJ (2007). Downregulation of death-associated protein kinase 1 (DAPK1) in chronic lymphocytic leukemia. Cell.

[39] Dunwell TL, Dickinson RE, Stankovic T, Dallol A, Weston V, Austen B, Catchpoole D, Maher ER, Latif F (2009). Frequent epigenetic inactivation of the SLIT2 gene in chronic and acute lymphocytic leukemia. Epigenetics.

[40] Claus R, Lucas DM, Stilgenbauer S, Ruppert AS, Yu L, Zucknick M, Mertens D, Buhler A, Oakes CC, Larson RA (2012). Quantitative DNA methylation analysis identifies a single CpG dinucleotide important for ZAP-70 expression and predictive of prognosis in chronic lymphocytic leukemia. J Clin Oncol.

[41] Chim CS, Pang R, Liang R (2008). Epigenetic dysregulation of the Wnt signalling pathway in chronic lymphocytic leukaemia. J Clin Pathol.

[42] Bennett LB, Taylor KH, Arthur GL, Rahmatpanah FB, Hooshmand SI, Caldwell CW (2010). Epigenetic regulation of WNT signaling in chronic lymphocytic leukemia. Epigenomics.

[43] Martinelli S, Kanduri M, Maffei R, Fiorcari S, Bulgarelli J, Marasca R, Rosenquist R (2013). ANGPT2 promoter methylation is strongly associated with gene expression and prognosis in chronic lymphocytic leukemia. Epigenetics.

[44] Kalinkova L, Sevcikova A, Stevurkova V, Fridrichova I, Ciernikova S (2023). Targeting DNA methylation in leukemia, myelodysplastic syndrome, and lymphoma: a potential diagnostic, prognostic, and therapeutic tool. Int J Mol Sci.

[45] Malik A, Shoukier M, Garcia-Manero G, Wierda W, Cortes J, Bickel S, Keating MJ, Estrov Z (2013). Azacitidine in fludarabine-refractory chronic lymphocytic leukemia: a phase II study. Clin Lymphoma Myeloma Leuk.

